# Supplementary figures and images for: Brain responsivity to emotional faces differs in men and women with and without a history of alcohol use disorder
Source: PLoS One. 2021 Jun 9;16(6):e0248831. doi: 10.1371/journal.pone.0248831 (PMC8189468; doi:10.1371/journal.pone.0248831)

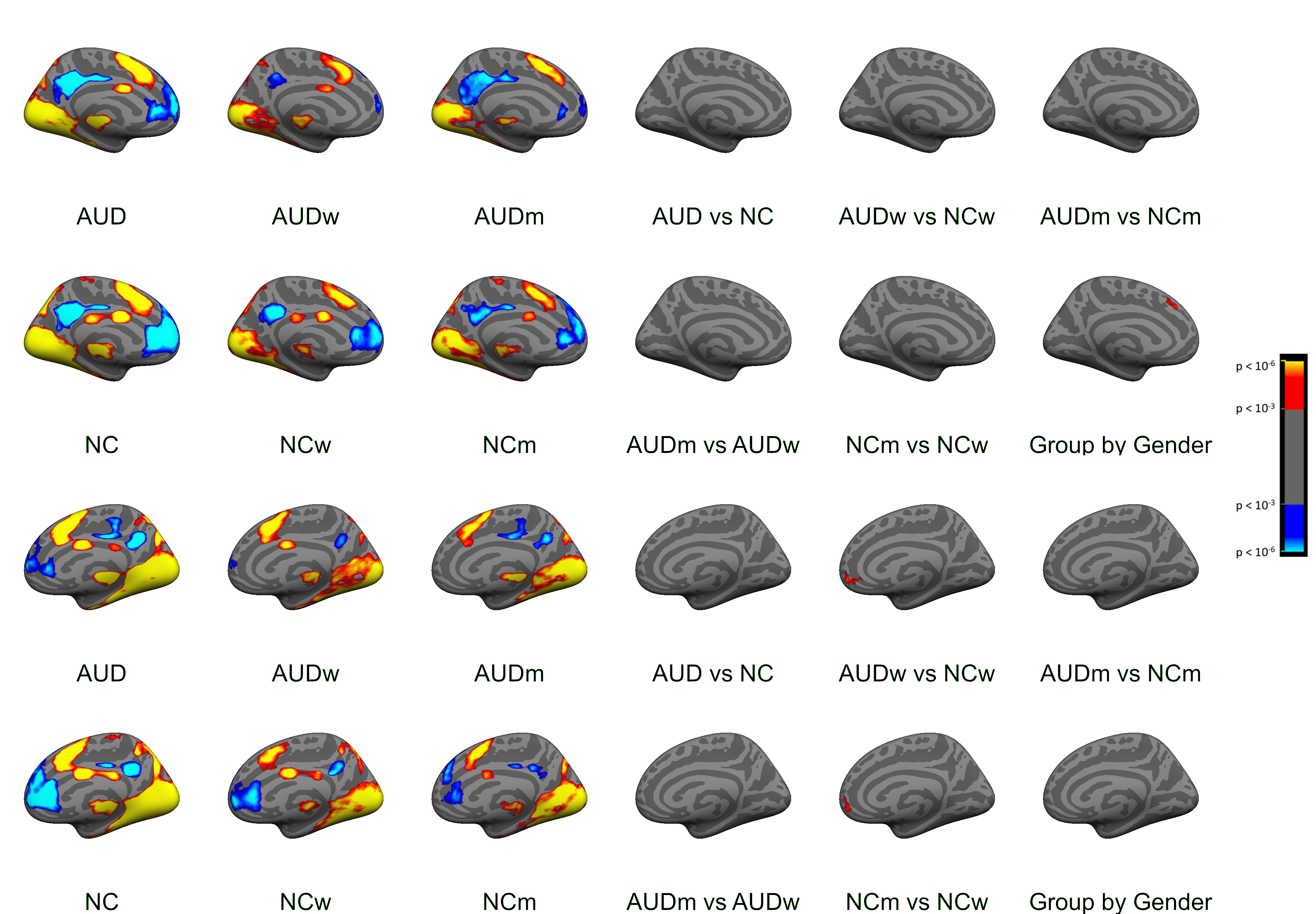

Supplement: S1 Fig — The left three columns show group maps, and the right three columns show group comparisons. The top two rows represent the left hemisphere, and the bottom two rows represent the right hemisphere. The clusters in this figure had a vertex wise threshold of p < .001 with a minimum cluster size of 100 mm2. This can result in more clusters being visible than are listed in Table 3 and S1 Table, wherein numbers were derived using permutation testing (cluster threshold p < .05, further corrected for analyses of left, right, and volume spaces). (PNG) [file pone.0248831.s001.png]

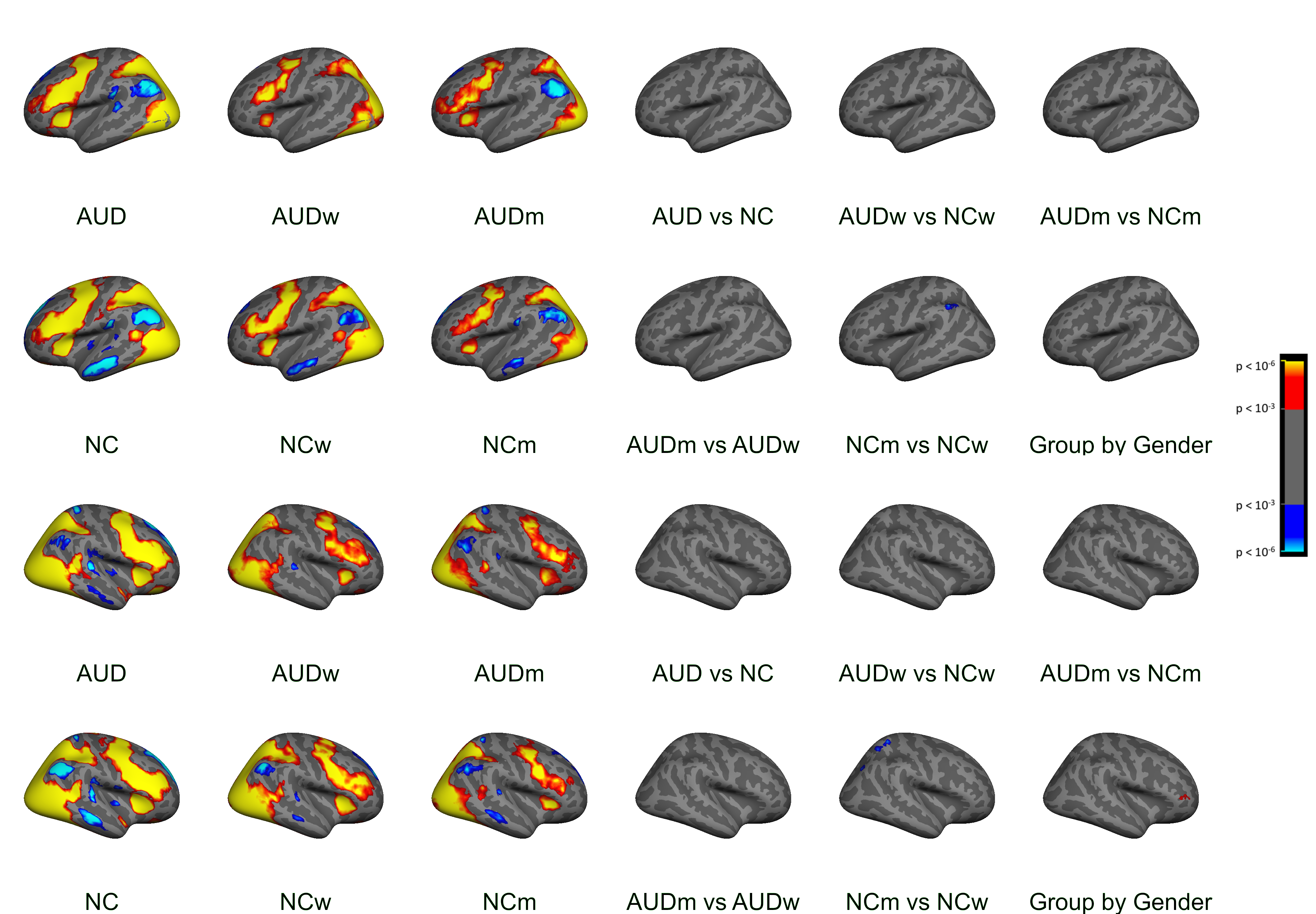

Supplement: S2 Fig — The left three columns show group maps, and the right three columns show group comparisons. The top two rows represent the left hemisphere, and the bottom two rows represent the right hemisphere. The clusters in this figure had a vertex wise threshold of p < .001 with a minimum cluster size of 100 mm2. This can result in more clusters being visible than are listed in Table 3 and S1 Table, wherein numbers were derived using permutation testing (cluster threshold p < .05, further corrected for analyses of left, right, and volume spaces). (PNG) [file pone.0248831.s002.png]

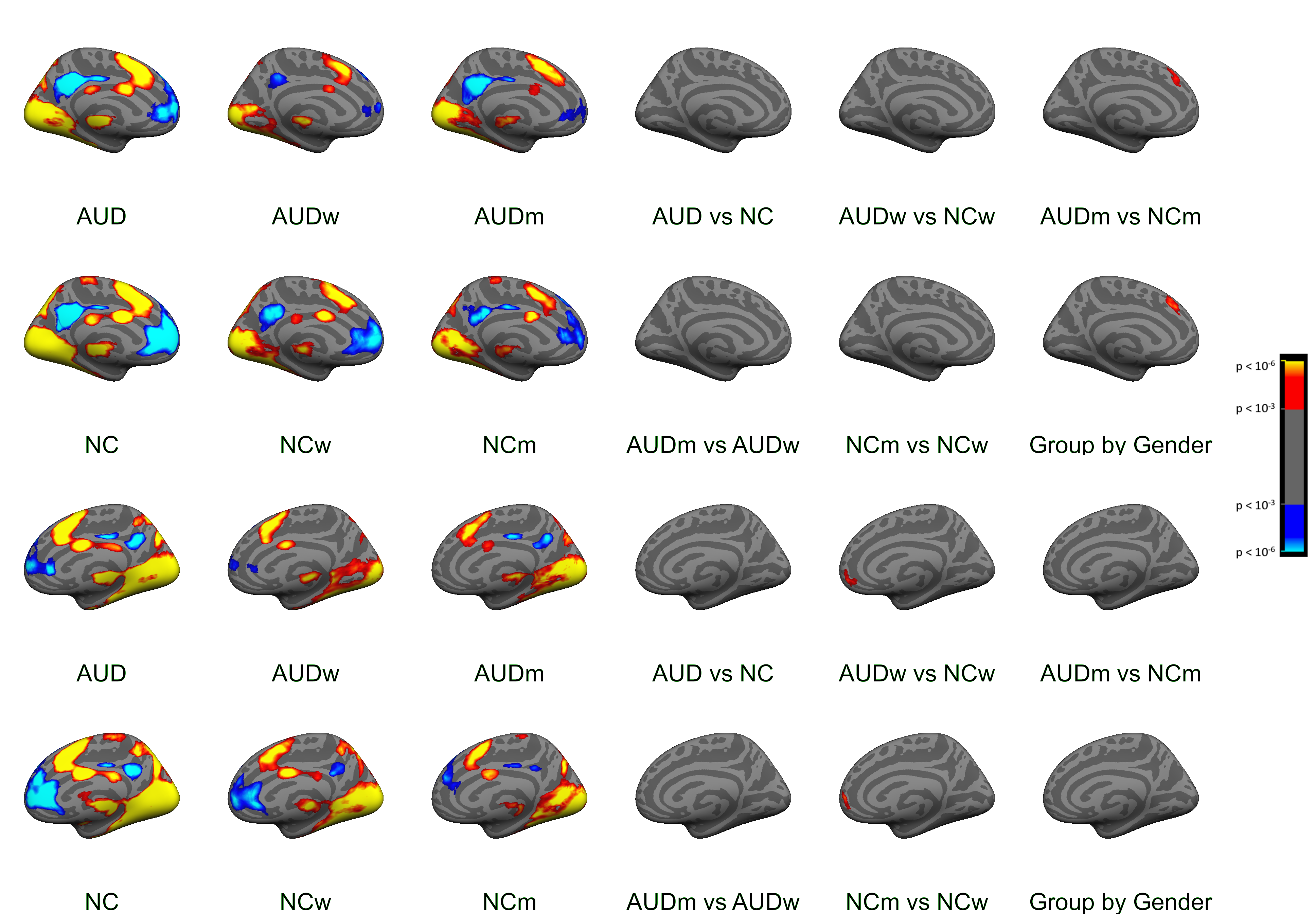

Supplement: S3 Fig — The left three columns show group maps, and the right three columns show group comparisons. The top two rows represent the left hemisphere, and the bottom two rows represent the right hemisphere. The clusters in this figure had a vertex wise threshold of p < .001 with a minimum cluster size of 100 mm2. This can result in more clusters being visible than are listed in Table 3 and S1 Table, wherein numbers were derived using permutation testing (cluster threshold p < .05, further corrected for analyses of left, right, and volume spaces). (PNG) [file pone.0248831.s003.png]

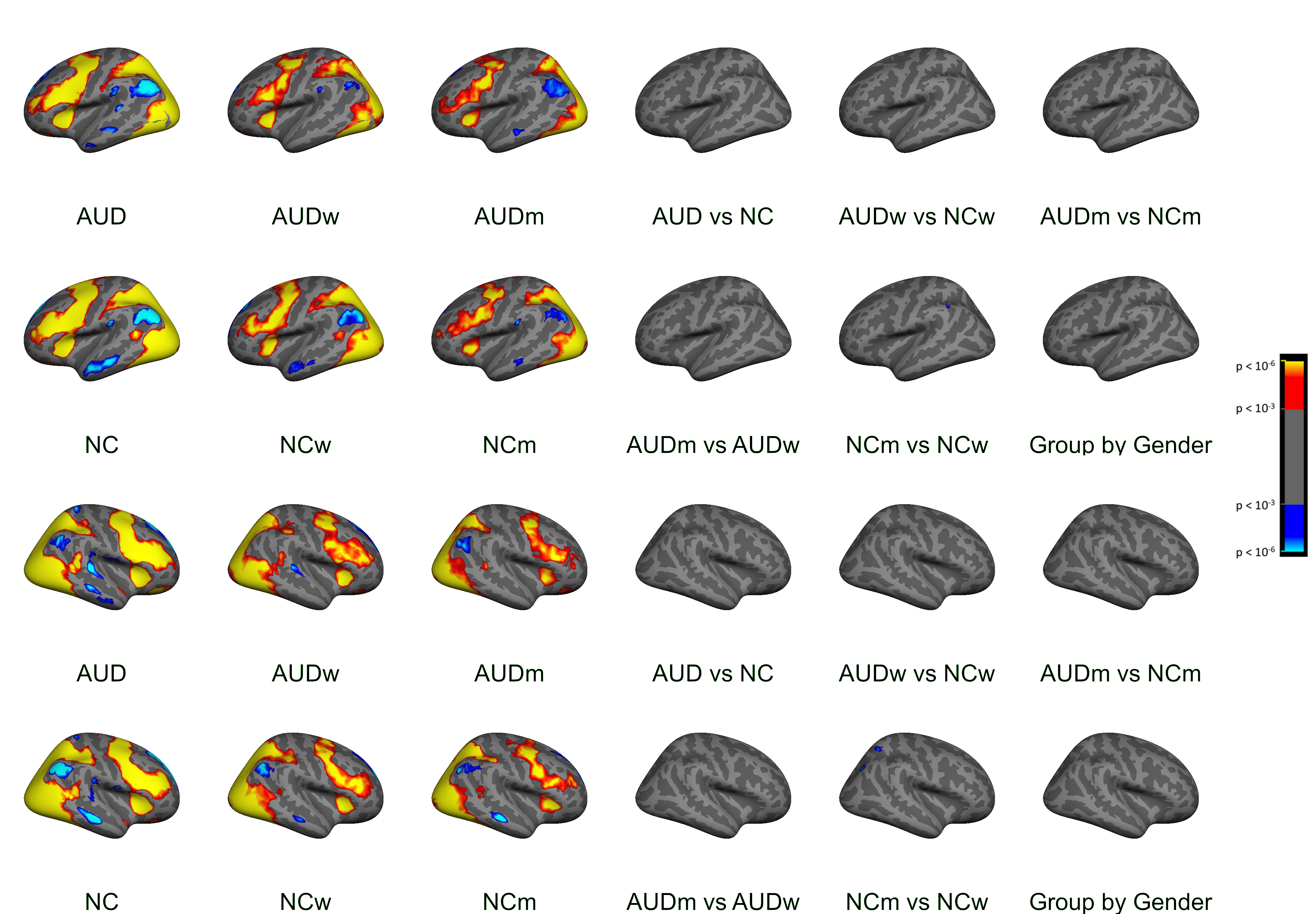

Supplement: S4 Fig — The left three columns show group maps, and the right three columns show group comparisons. The top two rows represent the left hemisphere, and the bottom two rows represent the right hemisphere. The clusters in this figure had a vertex wise threshold of p < .001 with a minimum cluster size of 100 mm2. This can result in more clusters being visible than are listed in Table 3 and S1 Table, wherein numbers were derived using permutation testing (cluster threshold p < .05, further corrected for analyses of left, right, and volume spaces). (PNG) [file pone.0248831.s004.png]

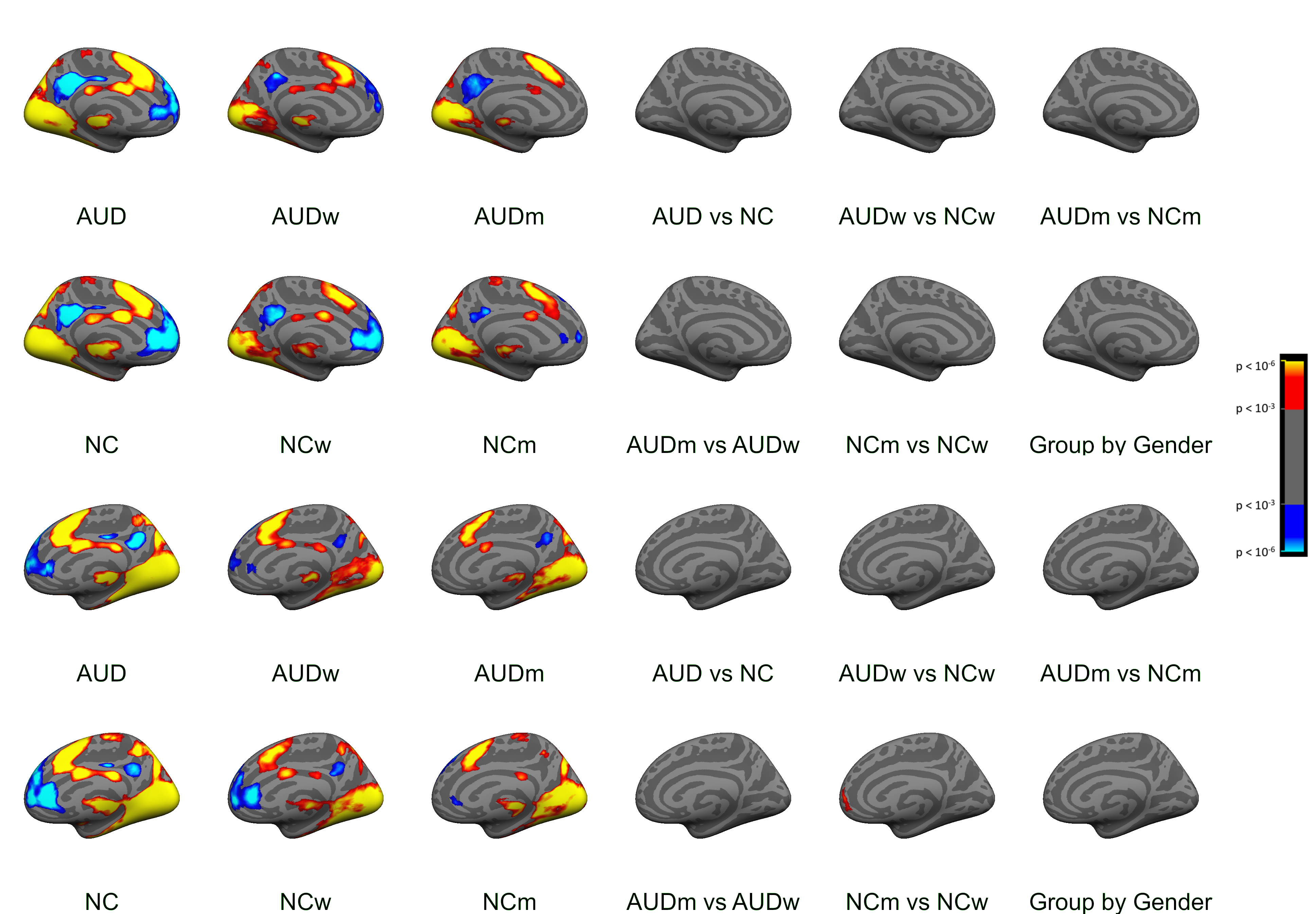

Supplement: S5 Fig — The left three columns show group maps, and the right three columns show group comparisons. The top two rows represent the left hemisphere, and the bottom two rows represent the right hemisphere. The clusters in this figure had a vertex wise threshold of p < .001 with a minimum cluster size of 100 mm2. This can result in more clusters being visible than are listed in Table 3 and S1 Table, wherein numbers were derived using permutation testing (cluster threshold p < .05, further corrected for analyses of left, right, and volume spaces). (PNG) [file pone.0248831.s005.png]

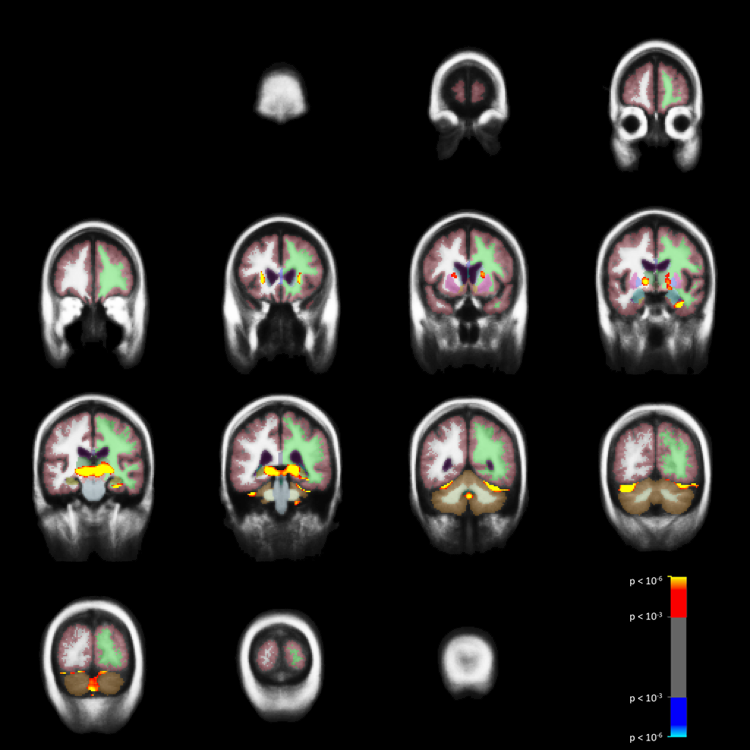

Supplement: S6 Fig — Cluster-corrected at p < .001 with minimum cluster size 300 mm3. Shown in neurological convention (left brain on the left side of the image). (PNG) [file pone.0248831.s006.png]

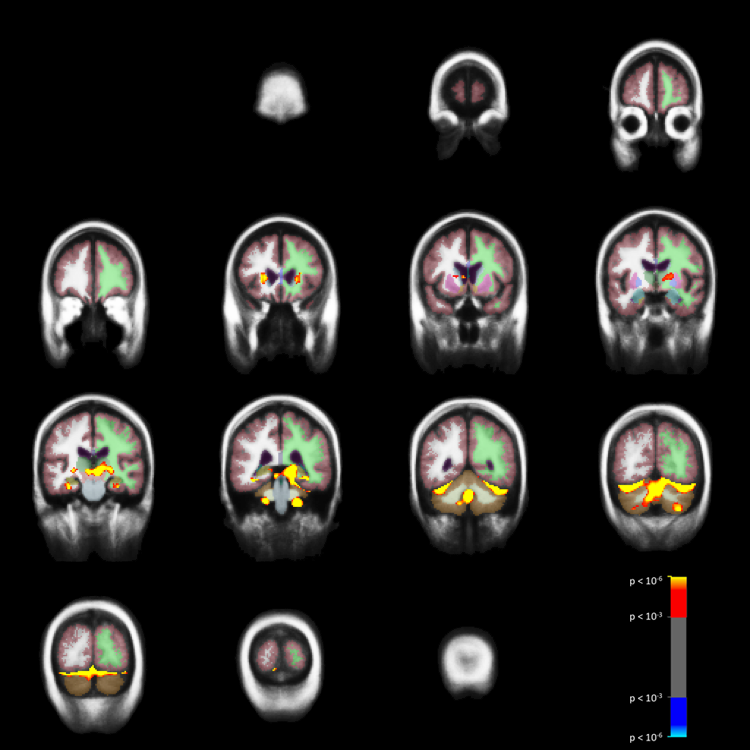

Supplement: S7 Fig — Cluster-corrected at p < .001 with minimum cluster size 300 mm3. Shown in neurological convention (left brain on the left side of the image). (PNG) [file pone.0248831.s007.png]

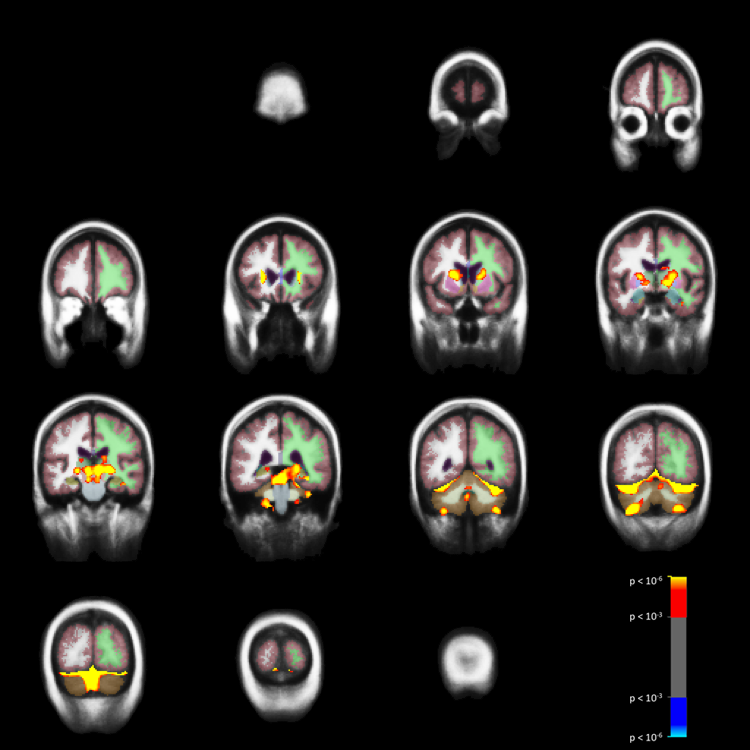

Supplement: S8 Fig — Cluster-corrected at p < .001 with minimum cluster size 300 mm3. Shown in neurological convention (left brain on the left side of the image). (PNG) [file pone.0248831.s008.png]

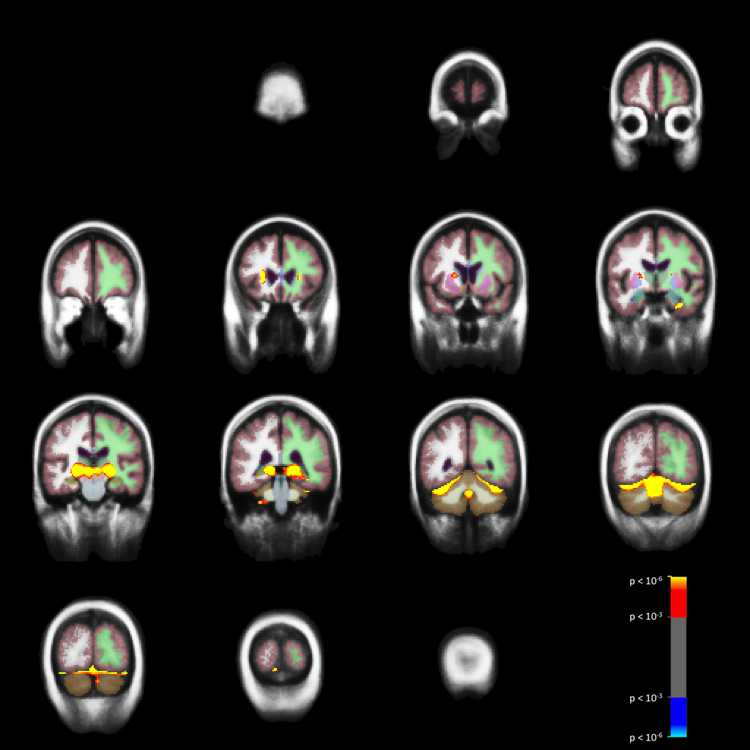

Supplement: S9 Fig — Cluster-corrected at p < .001 with minimum cluster size 300 mm3. Shown in neurological convention (left brain on the left side of the image). (PNG) [file pone.0248831.s009.png]

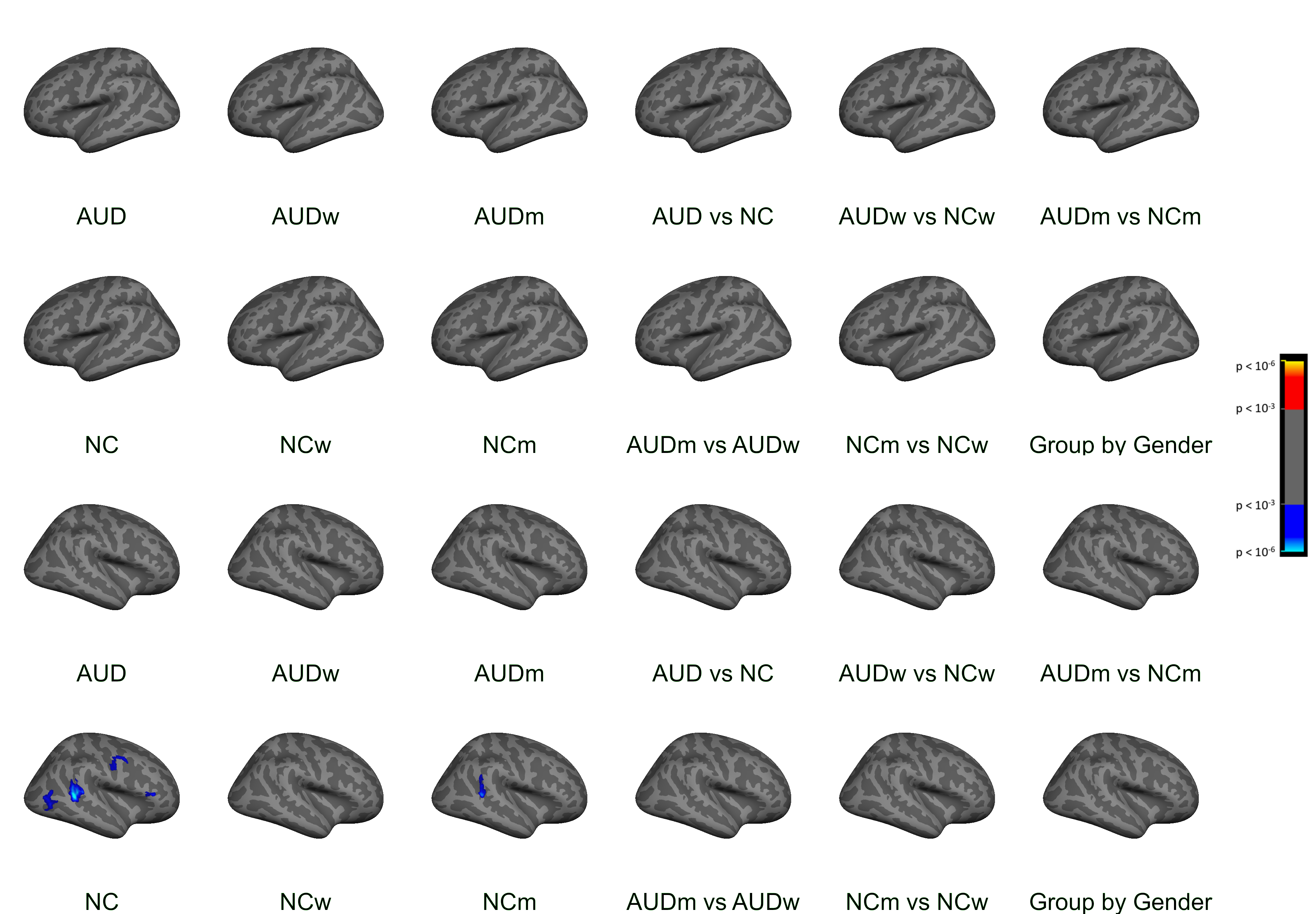

Supplement: S10 Fig — The left three columns show group maps, and the right three columns show group comparisons. The top two rows represent the left hemisphere, and the bottom two rows represent the right hemisphere. The clusters in this figure had a vertex wise threshold of p < .001 with a minimum cluster size of 100 mm2. This can result in more clusters being visible than are listed in Table 2, wherein numbers were derived using permutation testing (cluster threshold p < .05, further corrected for analyses of left, right, and volume spaces). (PNG) [file pone.0248831.s010.png]

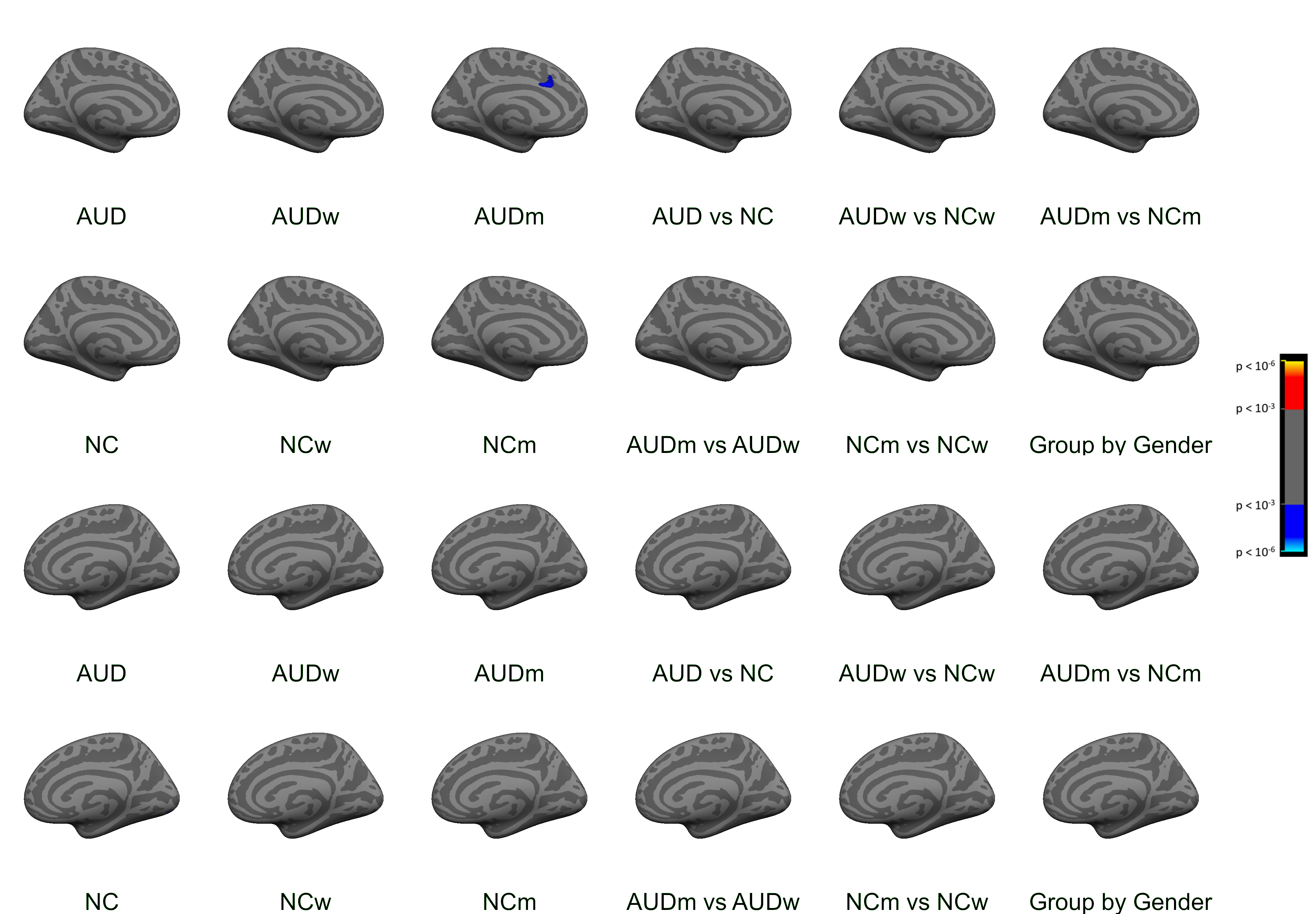

Supplement: S11 Fig — The left three columns show group maps, and the right three columns show group comparisons. The top two rows represent the left hemisphere, and the bottom two rows represent the right hemisphere. The clusters in this figure had a vertex wise threshold of p < .001 with a minimum cluster size of 100 mm2. This can result in more clusters being visible than are listed in Table 2, wherein numbers were derived using permutation testing (cluster threshold p < .05, further corrected for analyses of left, right, and volume spaces). (PNG) [file pone.0248831.s011.png]

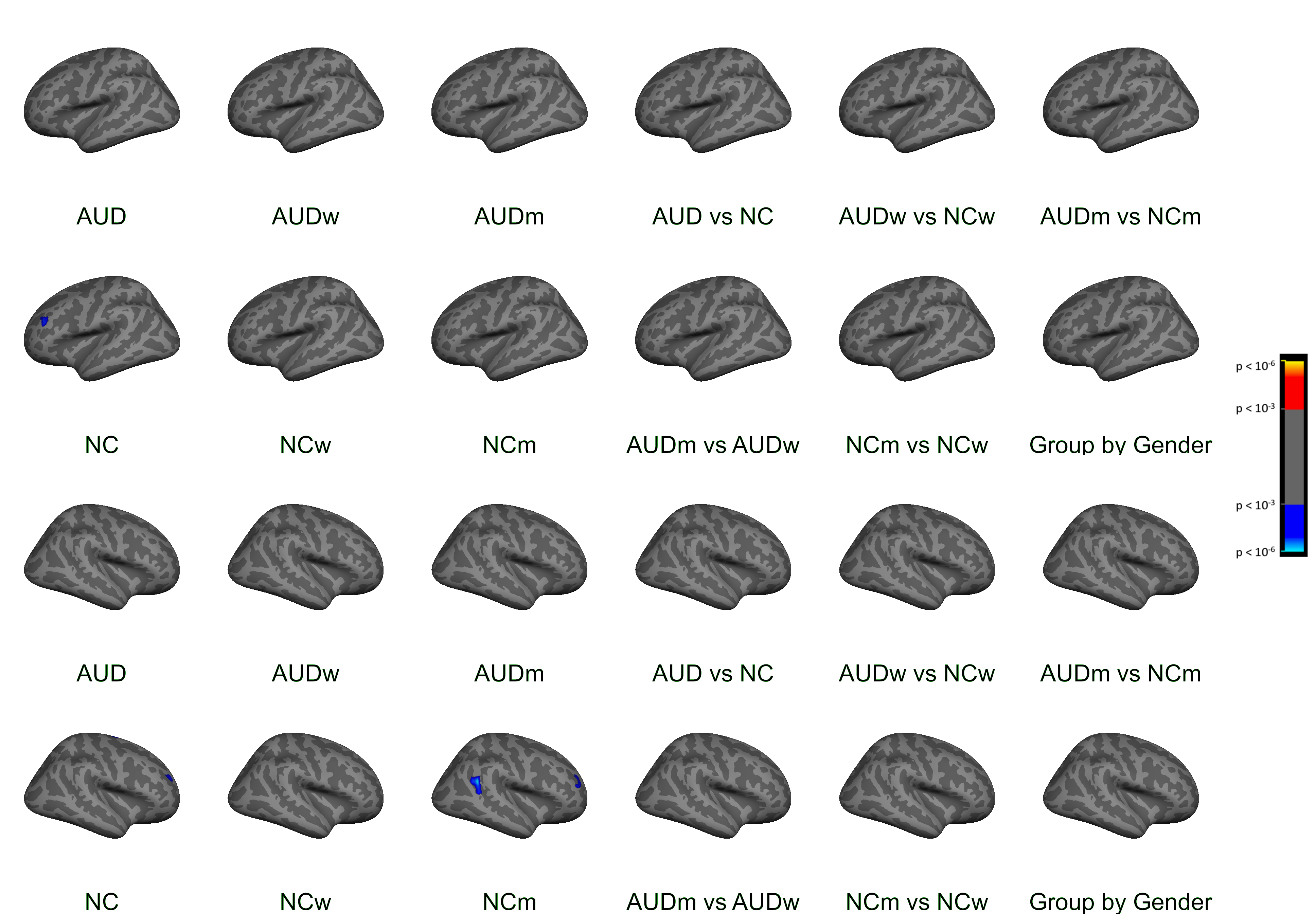

Supplement: S12 Fig — The left three columns show group maps, and the right three columns show group comparisons. The top two rows represent the left hemisphere, and the bottom two rows represent the right hemisphere. The clusters in this figure had a vertex wise threshold of p < .001 with a minimum cluster size of 100 mm2. This can result in more clusters being visible than are listed in Table 2, wherein numbers were derived using permutation testing (cluster threshold p < .05, further corrected for analyses of left, right, and volume spaces). (PNG) [file pone.0248831.s012.png]

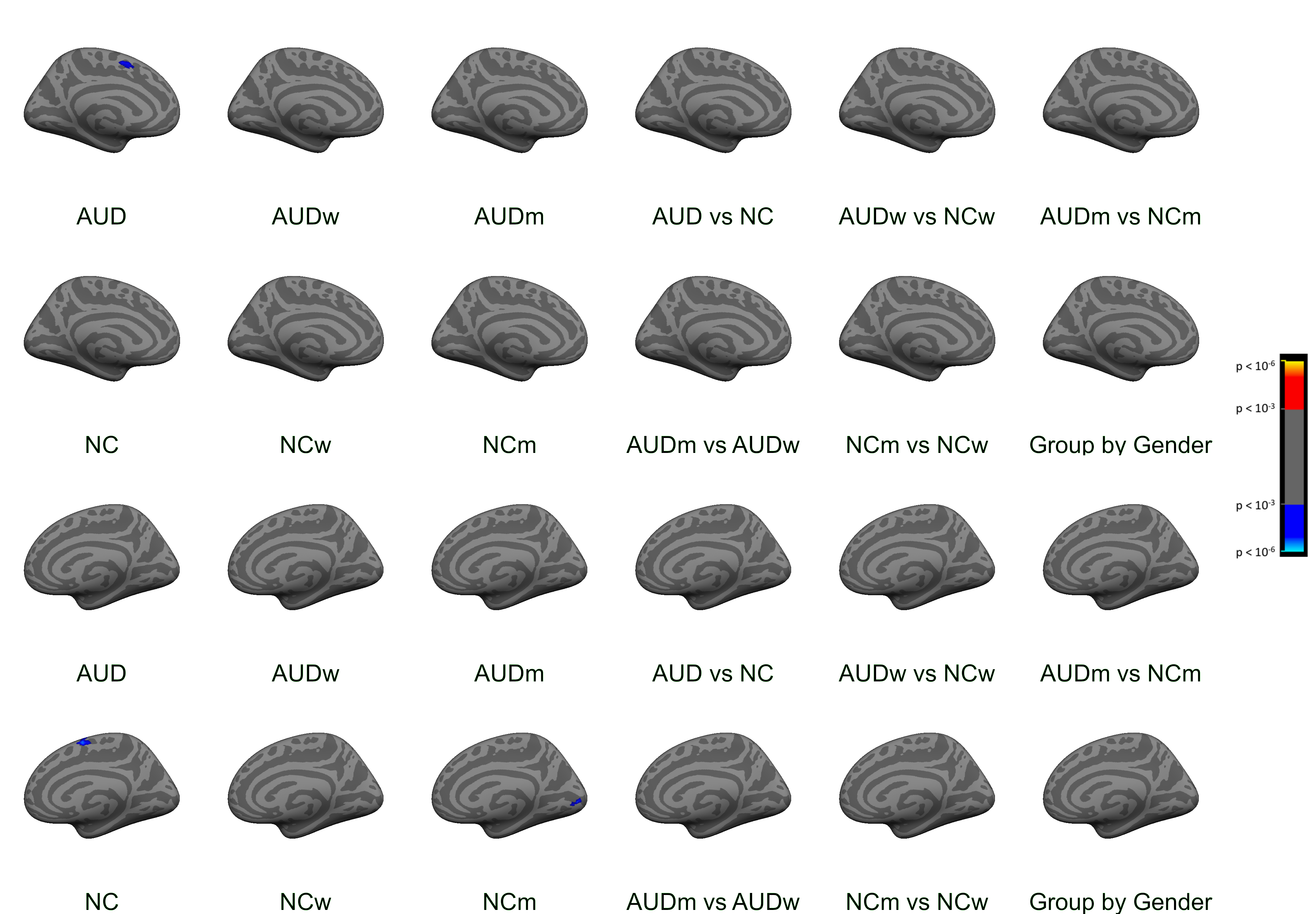

Supplement: S13 Fig — The left three columns show group maps, and the right three columns show group comparisons. The top two rows represent the left hemisphere, and the bottom two rows represent the right hemisphere. The clusters in this figure had a vertex wise threshold of p < .001 with a minimum cluster size of 100 mm2. This can result in more clusters being visible than are listed in Table 2, wherein numbers were derived using permutation testing (cluster threshold p < .05, further corrected for analyses of left, right, and volume spaces). (PNG) [file pone.0248831.s013.png]

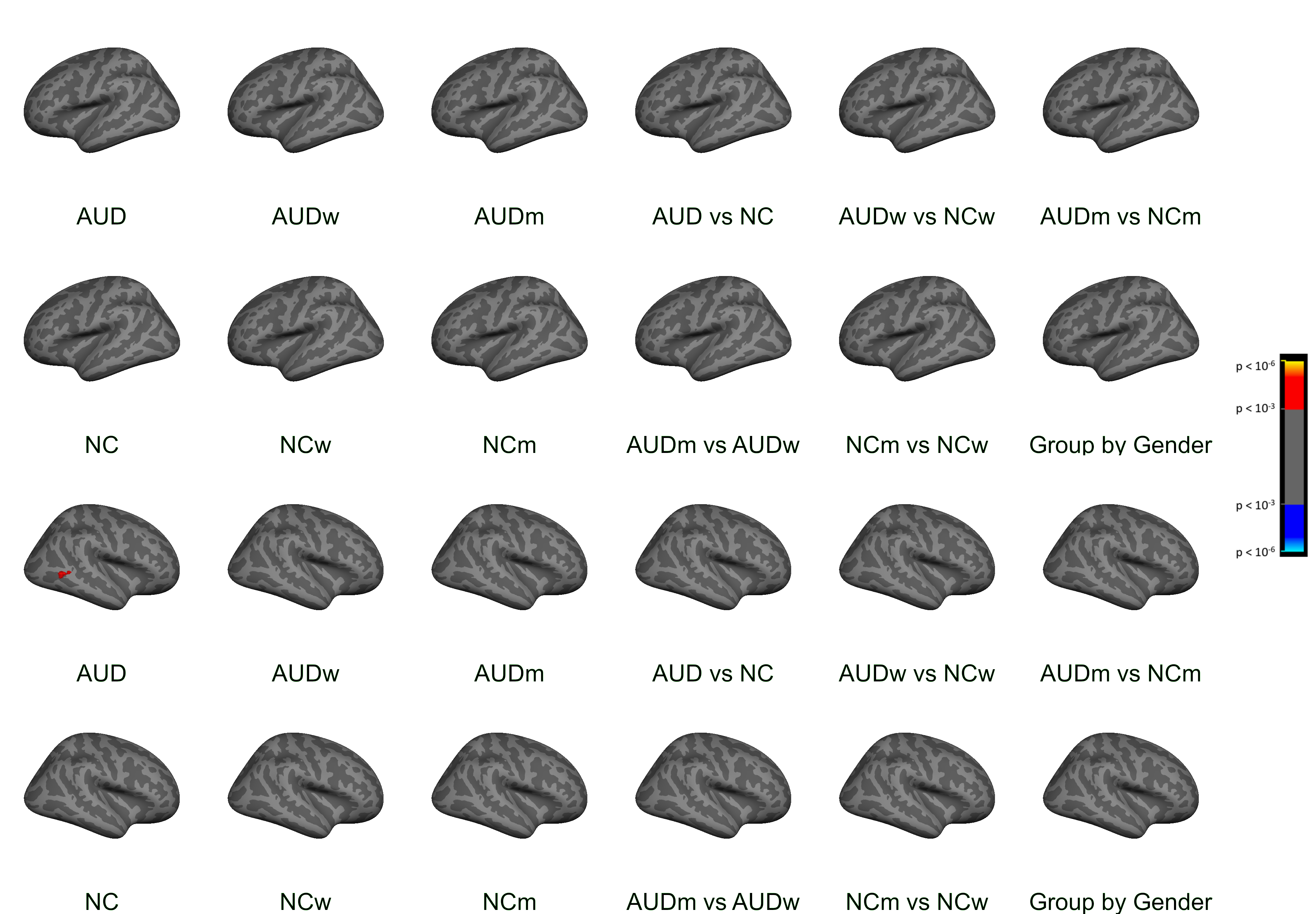

Supplement: S14 Fig — The left three columns show group maps, and the right three columns show group comparisons. The top two rows represent the left hemisphere, and the bottom two rows represent the right hemisphere. The clusters in this figure had a vertex wise threshold of p < .001 with a minimum cluster size of 100 mm2. This can result in more clusters being visible than are listed in Table 2, wherein numbers were derived using permutation testing (cluster threshold p < .05, further corrected for analyses of left, right, and volume spaces). (PNG) [file pone.0248831.s014.png]

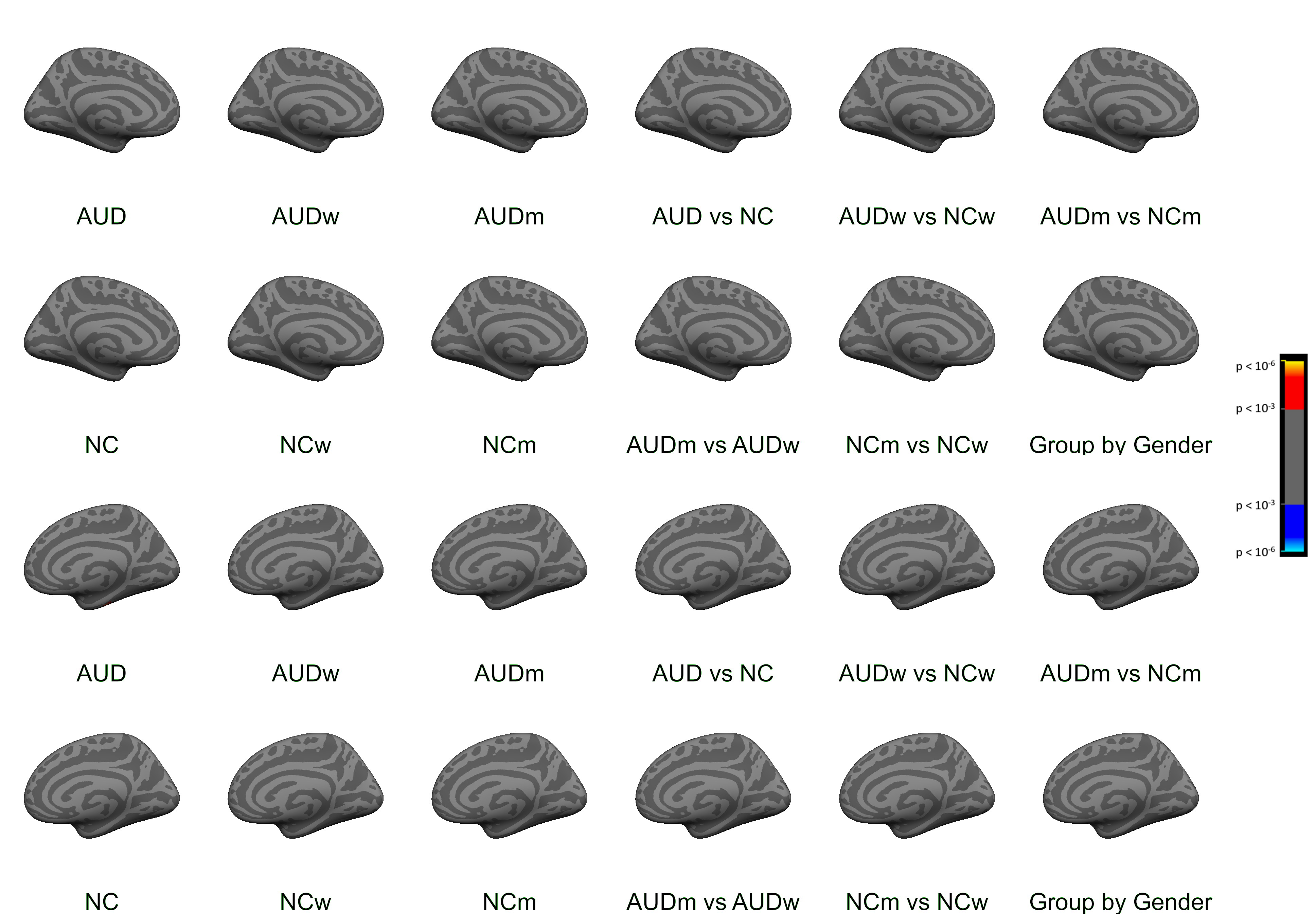

Supplement: S15 Fig — The left three columns show group maps, and the right three columns show group comparisons. The top two rows represent the left hemisphere, and the bottom two rows represent the right hemisphere. The clusters in this figure had a vertex wise threshold of p < .001 with a minimum cluster size of 100 mm2. This can result in more clusters being visible than are listed in Table 2, wherein numbers were derived using permutation testing (cluster threshold p < .05, further corrected for analyses of left, right, and volume spaces). (PNG) [file pone.0248831.s015.png]
