# Supplementary material for: Brain responsivity to emotional faces differs in men and women with and without a history of alcohol use disorder
Source: PLoS One. 2021 Jun 9;16(6):e0248831. doi: 10.1371/journal.pone.0248831 (PMC8189468; doi:10.1371/journal.pone.0248831)
Supplement: Attachment — Submitted filename: PONE-S-20-00533-R1-reply.docx [file pone.0248831.s019.docx]

**Kayle S. Sawyer, Ph.D.**

**Boston University School of Medicine, L-1004**

**72 East Concord Street**

**Boston, MA 02118**

July 23, 2020

Wi Hoon Jung, PhD

Academic Editor

*PLOS ONE*

Dear Dr. Jung:

We thank the editor and the reviewers for their valuable comments regarding our manuscript entitled “Brain Responsivity to Emotional Faces Differs in Men and Women with and without a History of Alcohol Use Disorder” (PONE-D-20-00671-R1). We also thank the editor and the reviewers for the effort and time they put into the review of the manuscript. We have addressed each comment below and revised the manuscript accordingly. We believe that the changes made in this revision have improved the quality of our manuscript.

**REVIEWER #1**

1. **Reviewer 1:** **The groups were well matched and the activation patterns were generally similar across the groups, lending assurance to replicability. The writing was clear and succinct.**

Authors: We appreciate the complimentary assessment.

2. **Reviewer 1: AUD subjects were abstinent for a minimum of 4 weeks but an average of 8 years. This wide range of sobriety supports persisting deficits in emotional and memory processing, but also in a heterogenous group, could length of sobriety have contributed to sex differences. Can length of sobriety be used as a co-variate in the analysis?**

Authors: We added text to the Limitations portion of the Discussion regarding sobriety with the following: “Regarding the variable of LOS, since there were no values for the NC group, the variable could not be used as a covariate in an analysis of group differences. However, we believe that the alcohol consumption and abstinence characteristics of our AUD cohort are representative of the national population [(World Health Organization 2019)](https://paperpile.com/c/zNbh3G/WQHt), thereby improving generalizability of our results.”

3. **Reviewer 1: Why was the Destrieux atlas used? Can some sort of justification be presented?**

Authors: We considered two atlases that were automatically provided with analyses conducted in FreeSurfer: the Destrieux atlas and the Desikan-Killiany atlas. We selected the more recent Destrieux atlas for its delineation between gyral and sulcal regions, thereby allowing us to more precisely pinpoint areas of activation.

We added the following phrase to the first sentence of the second paragraph of *Image Acquisition, Processing, and Statistical Analyses* in the Methods: “...and in order to delineate small regions distinguished by gyri and sulci, we used the Destrieux atlas [(Destrieux et al. 2010)](https://paperpile.com/c/afrII0/Ss92) for segmentation and parcellation.”

4. **Reviewer 1: AUDw had higher Hamilton depression Scale (HDS) scores than AUDm. Can the sex differences be accounted for by depression? Can the HDS score be used as a covariate?**

Authors: HRSD scores of 8, 16, and 25 or above indicate mild, moderate, or severe depression, respectively [(Zimmerman et al. 2013)](https://paperpile.com/c/zNbh3G/hu24). Because severe depression was an exclusion criterion for all of the participants, none of our participants scored over 18, and the average scores for all four subgroups (AUDm, AUDw, NCm, and NCw) were below 5, it is likely that depression contributed little to our observed gender differences.

We updated the *Participant Characteristics* section of the Results with the following text: “While AUDw had higher Hamilton Rating Scale for Depression scores than AUDm, the average scores for all four subgroups (AUDm, AUDw, NCm, and NCw) were low (below 5), so depression likely contributed little to our observed gender differences.”

5. **Reviewer 1: Positive faces elicited the least activation, as compared to both neutral and negative faces. Can the authors discuss this result in a bit more detail?**

Authors: We do not know why neutral faces might elicit greater activation than positive faces, and included the phrase, “While it is unclear why neutral faces would elicit higher activation than positive faces, a meta-analysis [(Fusar-Poli et al. 2009)](https://paperpile.com/c/zNbh3G/QD8h) showed that negative faces were widely reported to evoke strong activation.” We additionally reworked the first and second paragraphs of the Discussion to provide better context and interpretation regarding the stronger activation to negative faces.

**REVIEWER #2**

Introduction

Page 4:

1. **Reviewer 2: In paragraph 2 on Page 4, the authors transition from talking about the neurobiology underlying facial identity versus expression recognition in the previous paragraph to talking about gender differences in AUD. As a reader, I found this transition to be somewhat confusing and would suggest reframing paragraph 2 to begin by discussing the neurobiology underlying face and emotion processing and then transitioning to the potential implications for gender differences.**

Authors: As suggested, we reframed paragraph 2 to begin with brain regions associated with the encoding of face identity and emotional expressions. Then, as a transition to a discussion of gender differences, we added the following:

“Facial identity also has been examined in the context of gender [(Kret and De Gelder 2012)](https://paperpile.com/c/zNbh3G/mifk). For example, men have been reported to show stronger activation than women in several frontal and temporal brain regions [(Wrase et al. 2003; Fine et al. 2009)](https://paperpile.com/c/zNbh3G/H8FK+GvpJ), suggesting potential gender dimorphism in the processing of facial emotional expression. The gender differences in activation of these regions could be related to other factors observed to vary with emotion, such as hormones, genetics, and culture [(Kret and De Gelder 2012)](https://paperpile.com/c/zNbh3G/mifk). In addition to differentiating how facial emotion is processed, gender also influences the ways in which alcoholism impacts the brain, and further, how emotion, gender, and alcoholism interact [(Sawyer et al. 2019)](https://paperpile.com/c/zNbh3G/HipG).”

We also revised the wording of paragraph 3 to improve readability.

Page 4-5:

2. **Reviewer 2: The authors do not seem to offer any a priori hypotheses regarding gender differences in facial processing in AUD and only state that they wished to “characterize abnormalities in neural activation among abstinent participants with AUD.” I think that stating a clear hypothesis about what the authors expected to find would be helpful in terms of framing this study within the context of the current literature and guiding the subsequent analyses and discussion.**

Authors: In the Introduction, we have addressed this suggestion by describing differences between men and women, which includes the following explanation and subsequent hypothesis.

“When forming our specific a priori hypothesis with regard to AUD-related gender differences in response to emotional stimuli, we noted that the literature was mixed. Across brain regions, previous literature has reported abnormalities in multiple directions, with women evidencing greater brain activitivation in response to emotional stimuli, men having more brain activity, or having undetectable gender differences [(Verplaetse et al. 2020; Padula et al. 2015; Oscar-Berman et al. 2014; Nixon et al. 2014; Hoffman et al. 2019; Sawyer et al. 2019)](https://paperpile.com/c/zNbh3G/ueJh+Qdd9+Rtnr+aUca+sbx0+HipG). Based upon prior research in our laboratory, wherein brain regions of AUDw (compared to NCw) were found to be overactive in response to highly charged emotional stimuli [(Sawyer et al. 2019)](https://paperpile.com/c/zNbh3G/HipG) in the present study, we hypothesized that the brain responses to emotional faces by the AUDw would be hyperactive. Additionally, we expected to replicate prior results [(Marinkovic et al. 2009; Sawyer et al. 2019)](https://paperpile.com/c/afrII0/92Js+5vnk) showing widespread lower responses in AUDm than NCm.”

Although we observed gender differences, our hypotheses were not uniformly confirmed, and in the Discussion, we elaborated a possible explanatory factor for the AUD-related gender divergence: “Although we observed gender differences, our hypotheses were not uniformly confirmed. A possible explanation for the observed AUD-related gender divergence may be related to reports that women are more likely than men to be diagnosed with depression, anxiety disorders, and eating disorders [(Nolen-Hoeksema 2012)](https://paperpile.com/c/zNbh3G/ovjG). Therefore, alcohol consumption by AUDw might reflect a predisposition to regional hypo- or hyper-responsivity that increases the risk of depression, anxiety disorders, and eating disorders. In contrast, AUDm might consume alcohol to ‘let go’ of emotional inhibitions, thereby allowing themselves to experience their feelings. Whether cause or effect, AUD in men and women could become part of a vicious cycle which serves as a coping mechanism that also reinforces abnormalities in brain responsivity.”

Methods

Page 8:

3. **Reviewer 2: “The following a priori ROI were selected due to their previously established involvement in the emotion and face processing: amygdala, fusiform gyrus, hippocampus, parahippocampal gyrus, intraparietal sulcus, orbitofrontal cortex, superior temporal gyrus, and superior temporal sulcus.”**

**Did the authors correct for multiple comparisons across multiple a priori ROI’s? If not, could the authors clarify why they did not correct for multiple comparisons?**

Authors: We agree that multiple comparisons inflate the possibility of false positives (Type 1 error). To address this issue, we have updated the Results and added the following text to the Limitations, emphasizing the independence of hypotheses and also the increased possibility of false positive errors.

“... we conducted ROI analyses without applying corrections for multiple comparisons. Each ROI was selected as an individual hypothesis, because each of them was independently derived from results of previous literature. Therefore, we provided results for each ROI (all having been specified a priori), whether or not they were significant. However, this approach also could be considered in the context of a family of comparisons with an elevated false positive error rate.”

Page 8:

4. **Reviewer 2: “For surface analysis, the cluster threshold was set to 100 mm2 contiguous voxels of p < 0.001 for each contrast; for volume analysis, the minimum volume of contiguous voxels was 300 mm3.”**

**How were these thresholds determined? Using AFNI clustsim or a similar tool? Also, what do 100mm2 and 300 mm3 translate to in terms of voxels?**

Authors: As now noted in the Methods, 100 mm2 translates to roughly 200 vertices (the number is approximate due to the variability in vertex mesh density; fsaverage is approximately 2 vertices/mm^2^), and 300mm^3^ translates to 37.5 voxels at 8 mm3 (2mm isotropic). To address the question of threshold size, we added the following text to the Limitations:

“Based upon our qualitative judgement, we eliminated clusters smaller than an arbitrarily selected threshold of 100 mm^2^ (approximately 200 vertices) for cortical surface statistics, and 300 mm^3^ (37.5 voxels) for subcortical volume statistics. While these thresholds are likely to result in family-wise error rates above *p* = 0.05, we chose these limits in order to reduce the possibility of missing spatially-specific clusters. Consequently, we used a smaller (more lenient) cluster size threshold than might be suggested by cluster-thresholding methodological research [(Woo et al. 2014)](https://paperpile.com/c/zNbh3G/ZHkH). It should be noted, however, that cluster sizes that have been set by using a cluster-wise *p*-value may lead to higher underlying false positive rates than those expected when determining cluster size thresholds using Gaussian random field theory [(Eklund et al. 2016)](https://paperpile.com/c/zNbh3G/4Pxj). Therefore, instead of specifying a cluster-wise *p* value threshold, we used a specified fixed cluster-size threshold. Nevertheless, we did select a primary threshold of *p* < 0.001, which Woo and colleagues (2014) suggest “is a reasonable default for a range of typical cases.”

We also followed the suggestion of Woo and colleagues (2014), and added caveats regarding low spatial specificity to our captions in Fig 2, Table 2, and Table 3, as follows. “The clusters reported can be understood to span multiple functional regions [(Woo et al. 2014)](https://paperpile.com/c/zNbh3G/ZHkH). That is, they are not limited to a single region, as reported by the maximal vertex or voxel.”

Page 9:

5. **Reviewer 2: “For each participant, contrasts for the facial emotion conditions included: (1) positive faces vs. fixation, (2) negative faces vs. fixation, (3) neutral faces vs. fixation, (4) positive faces vs. negative faces, (5) positive faces vs. neutral faces, and (6) negative faces vs. neutral faces. Intergroup comparisons were: (1) AUD vs. NC, (2) AUDm vs. NCm, (3) AUDw vs. NCw, (4) AUDm vs. AUDw, (5) NCm vs. NCw, (6) men vs. women, and (7) Group by Gender interactions.**”

**For the cluster-level analyses, can the authors explain why they chose to implement individual t-tests for each contrast/intergroup comparison? In my view it would be simpler to model this as a 2 (Group: AUD vs NC) x 2 (Gender: Male vs Female) x 3 (Facial Expression: Positive, Negative, Neutral) repeated measures ANOVA and then submit any significant F-tests to post-hoc testing.**

Authors: We agree that the type of model suggested by the reviewer would be preferable. Indeed, we used such a model in our analyses of ROI results. However, when conducting vertex- and voxel-wise analyses to identify contiguous clusters, building an ANOVA model with cluster correction is not as simple as it is for an ROI analyses conducted with traditional statistical software. While identifying clusters with significant interaction effects is straightforward, it is not clear how to proceed with post-hoc comparisons. The cluster extents for the interaction F-tests and the clusters identified for the simple group comparison *t*-tests would be different, and we know of no neuroimaging software that provides a straightforward means of conducting vertex- and voxel-wise direct group comparisons within an interaction model, with respect to activation clusters. Considering this complication, we chose to conduct a more straightforward analysis for the vertex- and voxel-wise analyses — that is, to conduct the post-hocs directly. To address the possibility of false positives arising from these multiple comparisons, we added the following text to the Limitations:

“For our ROI results, we employed a traditional approach to statistical analyses, by examining interaction effects, followed by determining the significance of group differences. However, for the vertex-wise and voxel-wise analyses, in which we identified significant clusters of activation, there is no standard recommendation for how to combine group comparisons with interaction effects with respect to vertex or voxel clustering. Therefore, instead of using the traditional approach to assess the activation clusters we had hypothesized, we assessed the activation clusters for the group differences directly by using *t*-tests. This approach might engender additional comparisons, thereby possibly increasing Type I errors.”

6. **Reviewer 2: I would also recommend moving the supplemental methods section and supplemental Figure 1 to the main text to give more details regarding the MRI data collection, analysis, and the behavioral paradigm.**

Authors: As suggested, we transferred the Supplemental Methods section and Supplemental Figure 1 to the main text, thereby providing readers with earlier access to the details regarding the behavioral paradigm, and to the MRI data collection and analyses.

Results

Page 9:

7. **Reviewer 2: In the participants section, could the authors state whether there were any differences in alcohol quantity/frequency between males and females, particularly within the AUD group? I see that these differences are listed in Table 1, but it would be helpful to have these results in the main text.**

Authors: We added the following text to the Results: “The differences between AUDm and AUDw for QFI and LOS were not significant, but the DHD for the AUDm was significantly longer by 6.2 years compared to the AUDw.”

Page 10:

8. **Reviewer 2: “A different set of fixation-activated regions was more active during fixation than during the face conditions, forming the network known as the default mode network, because those regions typically are more active during rest than during attentionally- demanding cognitive tasks (Buckner et al., 2008). The regions making up this network include the superior frontal cortex, medial prefrontal cortex, medial temporal lobe structures, the middle temporal gyrus, the posterior cingulate cortex plus precuneus, and the angular gyrus.”**

**I would recommend removing this part regarding the default mode network as the main contrasts of interest primarily involve regions that are responsive to facial emotions and DMN findings are not discussed in the discussion section.**

Authors: We appreciate the reviewer’s suggestion, and we have removed the part regarding the default mode network.

Page 11-12:

9. **Reviewer 2: “The three clusters that were identified with significant group differences for contrasts between emotional face conditions had peak voxels contained within the left and right amygdala (872 mm3 and 384 mm3, respectively), and left hypothalamus (464 mm3).”**

**Could the authors clarify which clusters these are or display them in a figure? I do not see the any clusters listed as amygdala or hypothalamus in Table 3.**

Authors: Because Table 3 does not contain contrasts between emotional face conditions, we added a new Table 4, which clarifies the three clusters with peak voxels in those regions (for group comparisons). The three clusters reflect gender differences for NC participants (amygdala clusters), or gender differences for mixed NC and AUD participants (hypothalamus cluster), and as such, we believe they do not merit a figure. However, we did add figures to show clusters for group comparisons; see our reply to item 13 below for more detail.

Page 12-13:

10. **Reviewer 2: In the Neuroimaging Region of Interest Analyses section, did the intraparietal sulcus and/or the hippocampus show greater activation for faces relative to fixation within the ROI? If not, interpreting group differences may be difficult.**

Authors: The intraparietal sulcus did show substantially greater activation to the faces than fixation, but the hippocampus did not, as now noted with the following addition to the Results: “Overall, hippocampal activation in response to the encoded faces (vs. fixation) was negligible for all groups (Fig 4).” We also added text to more clearly indicate that the mean values we reported reflected differences relative to fixation.

In the Discussion, we added an interpretation of the intraparietal sulcus finding with the following addition: “...thereby supporting other evidence of working memory deficits in AUD populations (Oscar-Berman et al. 2014)” Regarding the hippocampus, as suggested by the reviewer, we emphasized that the lack of activation in the first place limited the interpretability of these results with the text, “... as compared to fixation, the activation of hippocampus to the encoded faces was negligible in all groups, which limits the interpretability of these results.”

11. **Reviewer 2: Finally- were there any group differences in behavior (either response times or accuracy) on the task? This would provide a bit more context in which to interpret the neuroimaging findings.**

Authors: We modified the text in the Limitations to more specifically acknowledge the absence of the behavioral measures of response times and accuracy in the present manuscript: “As noted in the Methods, analyses of the encoded face fMRI data reported in the present study were performed in the contexts of: (a) a task that included the influence of alcoholic and nonalcoholic beverage distractor cues, and (b) a memory task that measured participants’ response accuracies and reaction times to the probe face, but not to the encoded face. Therefore, interference from the distractor images may have influenced brain-activation or behavior. However, the distractor stimuli were equally distributed following each of the three types of emotional face valence conditions in order to ameliorate any differential impact of the distractors. Results obtained from the influences of distractor and probe stimuli on emotional processing will be presented in a future report.”

Discussion

Page 13-14:

12. **Reviewer 2: The first paragraph of the discussion seems to discuss the main task findings; while this is important information, I think it would be helpful to have a short paragraph at the very beginning of the discussion summarizing the results in order to provide the reader a road map for the discussion.**

Authors: We have added an initial paragraph as the “road map” requested by the reviewer. Based upon this new paragraph, we also added sub-headings to the Discussion.

Figures/Tables

13. **Reviewer 2: It would be helpful to have a figure showing some of the cluster-level group differences that are listed out in Table 3. In particular, it would be helpful to have a figure that not only shows each group but also directly displays the cluster-level group differences, particularly within the amygdala/hypothalamus (as mentioned in the Results section on Pages 11-12).**

Authors: For contrasts between emotions (positive vs. negative, positive vs. neutral, and negative vs. neutral), clusters for each group are presented in Table 2. For contrasts with fixation (positive vs. fixation, negative vs. fixation, and neutral vs. fixation), clusters for group comparisons are presented in Table 3. We had selected positive vs. fixation as an exemplar for presentation in Fig 2, because the negative vs. fixation and neutral vs. fixation contrasts yielded largely similar results. We expanded Fig 2 to directly display the cluster-level group differences. We added new supplemental figures, Fig S10 through S15, that show cortical surfaces for contrasts between emotional conditions. Like we did for Fig 2, we also expanded the supplemental figures to include the group differences. To clarify these figures, we added the following text to the legends of Table 2: “Fig S6 through Fig S9 show the corresponding subcortical cluster maps, and Fig S10 through Fig S15 show the corresponding cortical cluster maps.” and Table 3: “Fig 2 and Fig S1 through Fig S5 show the corresponding cortical cluster maps.”

We added a new Table 4 that addresses the contrasts between emotions (positive vs. neutral, negative vs. neutral, and positive vs. negative), wherein group comparisons yielded significant clusters that included the amygdala and hypothalamus. These results pertain to gender differences in the NC group for the amygdala, and gender differences in the larger group of combined AUD and NC participants for the hypothalamus.
