# Supplementary material for: Brain responsivity to emotional faces differs in men and women with and without a history of alcohol use disorder
Source: PLoS One. 2021 Jun 9;16(6):e0248831. doi: 10.1371/journal.pone.0248831 (PMC8189468; doi:10.1371/journal.pone.0248831)
Supplement: Attachment — Submitted filename: PONE-S-20-00533-R2-reply.docx [file pone.0248831.s020.docx]

**Kayle S. Sawyer, Ph.D.**

**Boston University School of Medicine, L-1004**

**72 East Concord Street**

**Boston, MA 02118**

January 25, 2021

Wi Hoon Jung, PhD

Academic Editor

*PLOS ONE*

Dear Dr. Jung:

We thank the editor and the reviewers for their valuable comments regarding the revision to our manuscript entitled “Brain Responsivity to Emotional Faces Differs in Men and Women with and without a History of Alcohol Use Disorder” (PONE-D-20-00671-R2). We also thank the editor and the reviewers for the effort and time they put into the second review of the manuscript, and their accommodation throughout this submission. We have addressed each comment below and revised the manuscript accordingly. We believe that the changes made in this revision have improved the manuscript.

**REVIEWER #1**

Previous Review: AUD subjects were abstinent for a minimum of 4 weeks but an average of 8 years. This wide range of sobriety supports persisting deficits in emotional and memory processing, but also in a heterogenous group, could length of sobriety have contributed to sex differences. Can length of sobriety be used as a co-variate in the analysis?

Previous Authors’ Reply: We added text to the Limitations portion of the Discussion regarding sobriety with the following: “Regarding the variable of LOS, since there were no values for the NC group, the variable could not be used as a covariate in an analysis of group differences. However, we believe that the alcohol consumption and abstinence characteristics of our AUD cohort are representative of the national population (World Health Organization 2019), thereby improving generalizability of our results.”

This variable (LOS) be used as a covariate among the AUD group only as a post hoc test to determine if it modulates connectivity.

**AUTHORS:** In this paper, we did not perform any analyses of connectivity. The aims of the present study were to explore how gender and AUD interact to influence emotional processing and memory (as indicated by brain activation in response to the task). Examination of connectivity was beyond the scope of this report. In case Reviewer 1 was referring to the relationship of LOS to “activation,” we restructured the final paragraph in the Limitations section, and included a phrase regarding the use of LOS as a covariate. The paragraph now reads as follows:

“Finally, our AUD subjects were abstinent for a minimum of four weeks but an average of 8.3 years. This wide range of sobriety speaks to the persistent nature of emotion processing deficits in AUD populations, and whether such deficits recover differently over the course of short- and long-term abstinence in men and women [13]. Moreover, we believe that the alcohol consumption and abstinence characteristics of our AUD cohort are representative of the national population [36], thereby improving generalizability of our results. Additionally, the topic of persistence vs. recovery remains a promising direction for future studies, for example, with analyses of brain activity in relation to length of sobriety and gender for the AUD group [82,83]. The average length of sobriety was longer for AUDm than for AUDw, which might have influenced the gender differences we observed. Since there were no abstinence values for the NC group, the variable could not be used as a covariate in an analysis of group differences.”

**REVIEWER #2**

The authors have largely responded to my reviews satisfactorily and I believe this paper makes an interesting contribution to the literature. However, I still have a couple of points of concern regarding the analysis.

i) In their response letter, the authors state that when they chose a fixed arbitrary cluster-size threshold, at least in part because “cluster sizes that have been set by using a cluster-wise p-value may lead to higher underlying false positive rates than those expected when determining cluster size thresholds using Gaussian random field theory (Eklund et al. 2016).”

The authors should consider using mri_glmfit-sim (or an equivalent program) to provide the cluster size threshold at an initial p-value of p=.001 that would be equivalent to a cluster corrected p<.05 or provide the equivalent p-value for a cluster corrected threshold with an initial p-value of p=.001 and a cluster size of 100 mm^2^. It is my understanding that the faulty assumptions in this procedure leading to the Eklund paper have since been fixed within most neuroimaging software packages (Greve and Fischl, 2017; Cox, Chen, Glen, Reynolds, and Taylor, 2017). Although I am sympathetic to the view that an alpha level of p<.05 is an arbitrary threshold and it should be carefully considered whether or not to actually use an alpha level of p<.05, readers should have some quantitative point of reference for judging the significance of the clusters reported.

**AUTHORS:** We agree completely that the Reviewer’s suggested approach of cluster alpha thresholding is better than the simple cluster size thresholding we had used previously, and consequently, we re-analyzed all of the clusters using the permutation testing procedure included with mri_glmfit-sim. This resulted in the removal of many of the clusters previously presented in Table 3, along with the entirety of Table 4. We edited the Abstract, Results, and Discussion to reflect the remaining clusters.

Regardless of the threshold or correction technique employed, large clusters can be interpreted with greater confidence, while smaller clusters have a higher chance of Type I error. However, using a stricter threshold eliminates reporting of the smaller clusters. We added text to the Methods and Limitations to reflect the balancing of Type I and Type II error levels.

**REVIEWER #2**

ii) With regard to conducting F-tests for cluster-level analyses, the authors state in their response letter that “building an ANOVA model with cluster correction is not as simple as it is for an ROI analyses conducted with traditional statistical software... The cluster extents for the interaction F-tests and the clusters identified for the simple group comparison t-tests would be different, and we know of no neuroimaging software that provides a straightforward means of conducting vertex- and voxel-wise direct group comparisons within an interaction model, with respect to activation clusters.”

I would suggest conducting a whole-brain repeated measures 2 (Group: AUD vs NC) x 2 (Gender: Male vs Female) x 3 (Facial Expression: Positive, Negative, Neutral) ANOVA on the BOLD response data. Then, using each individual significant cluster for each interaction effect (Group-by-Gender, Group-by-Facial Expression, Gender-by-Facial Expression, Group-by-Gender-by-Facial Expression), extract the average parameter estimates for each within-subject condition (in this case, each of the three levels of facial expression) for each subject, and then import these data into SPSS (along with the data for the between-subjects variables) where post-hoc testing for interaction effects within a repeated measures model can be conducted.

I have been able to conduct this procedure successfully in SPM and AFNI in the past; is there not a parallel way to do this in Freesurfer? While this is not a perfect procedure, I believe that it does somewhat control for Type I error since it limits the amount of individual contrasts tested for in the main neuroimaging analysis and it allows for the directionality of the interaction to be probed.

**AUTHORS:**

We appreciate the detailed explanation, and now understand the Reviewer’s suggested approach, as afforded by SPM and AFNI. In FS-FAST, the standard approach is to calculate first-level contrasts, then calculate each group comparison separately. We had constructed the interaction effect group comparison using this page as a guide: <https://surfer.nmr.mgh.harvard.edu/fswiki/Fsgdf4G0V#Contrast7gender-x-handedness.mtx>

For the present manuscript, we revised and updated the Limitations as follows:

“For our ROI results, we employed a traditional approach to statistical analyses, by examining interaction effects, followed by determining the significance of group differences. Instead of using a factorial ANOVA to assess the significant activation clusters with subsequent within-cluster post-hocs (that is, combining group comparisons with interaction effects for vertex-wise and voxel-wise analyses), we assessed the activation clusters for the group differences directly across all vertices and voxels. Even though this approach engenders additional comparisons and increases the likelihood of making Type I errors, it has the benefit of identifying clusters in locations other than those where interactions are present. We used a primary (vertex- or voxel-wise) threshold of *p* < 0.001 for each group comparison. By considering these analyses to be a family of seven comparisons, the Bonferroni-adjusted family-wise primary threshold would be *p* < 0.007.”

Again, we appreciate the suggestions of the reviewers, and especially this year, we are grateful for the patience of everyone involved.

Sincerely,

Kayle Sawyer
